# Current landscape of BRAF-V600E metastatic CRC management in Latin America: an expert Latin American panel’s recommendations

**DOI:** 10.3332/ecancer.2024.1807

**Published:** 2024-12-04

**Authors:** Anelisa K Coutinho, Yazmin Carolina Blanco Vazquez, Markus Andret Cavalcante Gifoni, Angela Marie Jansen, Juan Manuel O’Connor, Juan Carlos Samamé Pérez-Vargas, Mariana Rico-Restrepo, Gayatri Sanku, Guillermo Mendez

**Affiliations:** 1 Clínica AMO, DASA, Salvador, Bahia, Brazil; 2 Centro Médico ABC Observatorio, Ciudad de Mexico 01120, Mexico; 3 Universidade Federal do Ceara, Fortaleza 60430-160, Brazil; 4 Oncologia D’Or, Fortaleza 60135-237, Brazil; 5 Americas Health Foundation, Washington, DC, USA; 6 Instituto Alexander Fleming, Buenos Aires, Argentina; 7 Clínica San Felipe, Lima, Peru; 8 Hospital Nacional Arzobispo Loayza, Lima 15072, Peru; 9 Americas Health Foundation, Bogota, Colombia; 10 Hospital de Gastroenterologia ‘Carlos Bonorino Udaondo’, Buenos Aires 1264, Argentina; a https://orcid.org/0000-0003-2988-0446; b https://orcid.org/0000-0003-1269-4567; c https://orcid.org/0000-0003-2980-2421; d https://orcid.org/0000-0002-6975-5466; e https://orcid.org/0000-0001-7818-8670; f https://orcid.org/0000-0001-7234-0897; g https://orcid.org/0009-0002-8550-957X

**Keywords:** mCRC in Latin America, metastatic colorectal cancer, precision medicine, V600E-BRAF mCRC, V600E-BRAF-mutation

## Abstract

Colorectal cancer is the second leading cause of cancer death in Latin America (LA) with a projected 65.4% increase by 2040. Up to 10% of metastatic CRC (mCRC) patients in LA had an activating BRAF mutation. In clinical trials, targeted therapies for BRAF-V600E mutated mCRC have improved patient outcomes. However, in LA, BRAF-V600E testing and treatment of positive patients remains variable. To address this need, the Americas Health Foundation convened a meeting of LA experts on *BRAF-V600E* mCRC to develop treatment recommendations. The expert panel addressed the current landscape of BRAF-V600E mCRC testing, diagnosis and treatment in the region and identified significant limitations. Local guidelines, multidisciplinary boards, and tumor genotyping are among the recommendations. The panel also made first-line, second-line and surgery recommendations for patients after diagnosis.

## Background

*BRAF-V600E* mutated metastatic colorectal cancer (mCRC) (*BRAF-V600E* mCRC) impacts patients globally; Latin America (LA) has a 4%–12% prevalence. The v-RAF murine sarcoma viral oncogene homolog B1 protein (BRAF) is the primary kinase in the RAS/RAF/mitogen-activated protein kinas (MEK)/MAPK intracellular-signaling pathway regulating the cell cycle. The *BRAF-V600E* mutation causes constitutive kinase activation, uncontrolled cell division and neo-angiogenesis and metastases [1]. *BRAF* mutations are found in up to 10% of patients with mCRC and 95% are *V600E* [2]. While chemotherapeutics are available for patients with mCRC [3], patients with *BRAF V600E*-mutated mCRC have an 11–19 month median overall survival (OS), indicating a therapeutic gap in the efficacy of standard mCRC therapies. Recently, novel therapeutics have improved *BRAF*-mutated mCRC outcomes for patients globally. However, delayed regulatory approvals and affordability compromise treatment access in LA. This paper describes the landscape of *BRAF-V600E* mCRC therapy in LA and suggests ways to improve treatment and patient outcomes.

## Methods

AHF used PubMed, MEDLINE and EMBASE to identify LA-based oncologists who have published in colorectal cancer (CRC), oncology and *BRAF* testing since. AHF used the following search terms: *‘BRAF*,’ *‘BRAFV600,’* ‘molecular testing’ and ‘ CRC’ in combination with ‘ LA ‘ from 1 January 2017 to 10 June 2022. The identified articles were in English, Portuguese and Spanish. They met on 28, 29 November and 1 December 2022, to examine the landscape of diagnosing and treating *BRAF-V600E* CRC in LA and to create recommendations for optimum diagnosis and treatment. AHF assigned each panel member a question on *BRAF-V600E* CRC in LA. Individual panel members answered questions based on the AHF literature review, their reviews and personal knowledge. The panel reviewed and amended each answer during a 3-day meeting with many discussion rounds. Following the meeting, the panel evaluated and approved the final document.

## Results

### Epidemiology of mCRC

According to GLOBOCAN, CRC is the third most common cancer, and the second largest cause of death in LA and the Caribbean, with 186.5 cases and 86.5 deaths per 100,000 people. Alarmingly, LA CRC incidence is expected to rise by 65.4% by 2040 [1].

*BRAF*-*V600E* is seen in over 95% of *BRAF-*mutated mCRC patients and is female-associated, often right-sided and advanced, mucinous, with deficient mismatch repair (dMMR) and serrated adenoma pathway mutation [2, 4–6]. Among a cohort of 1,742 patients diagnosed with CRC in Argentina, Brazil, Chile, Mexico, Paraguay, Peru and Puerto Rico, the prevalence of *BRAF*-*V600E* mutation was 47.8% [7]. In a meta-analysis of *BRAF-V600E* mutation frequency across LA, variations in frequency were found by region (4.0%–12.2%), underscoring the importance of detecting and managing this form of mCRC in LA [8].

### Understanding BRAF-mutational status in LA

**Figure 1.**
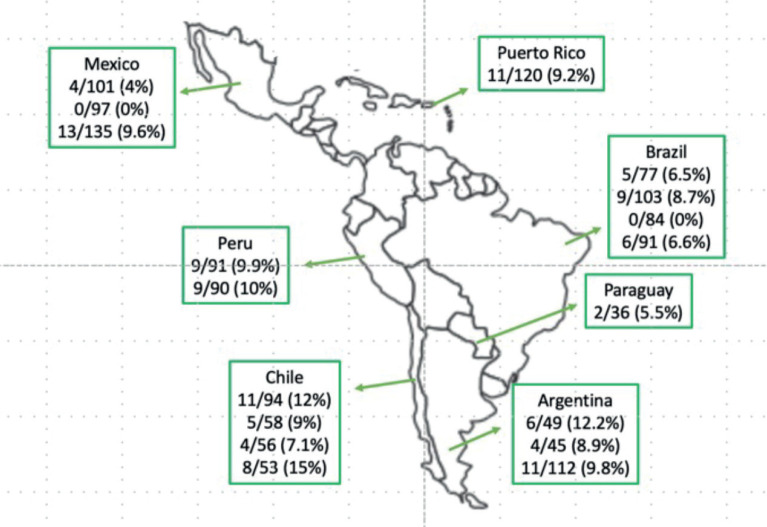
BRAF-V600E CRC prevalence in LA. Data taken from published studies [9–18].

*BRAF-V600E* mCRC cases are increasing in prevalence and mortality across LA. In Mexico, scientists discovered regional differences in *BRAF-V600E* tumors (Western Mexico 4%, Northeast Mexico 0% and Central Mexico 9.6%) [9, 10]. *BRAF-V600E* mCRC frequency has also been studied in Argentina (12.2%) [11], Brazil (6.5%, 8.7% and 6.6%) [12–14], Chile (9% and 12%) [15, 16] and Peru (9.9% and 10%) [17, 18]. Regionally, there are still gaps in the detection and mutational analysis of *BRAF-V600E* [19] (Figure 1).

To prescribe the optimal treatment, the *RAS/BRAF*-mutational status must be determined by either a tumor biopsy (primary or metastatic) or a less invasive liquid biopsy (LB) if no tumor material is available [20]. The advantages of liquid-based molecular profiling include studying intra- and inter-tumor genomic heterogeneity and performing tumor profiling without tissue.

Without local guidelines on screening, treating and monitoring *BRAF-V600E* mCRC, oncologists in LA often use international standards for testing and treatment decisions. The Argentine Association of Clinical Oncology recommends performing *RAS*, *BRAF* and MMR/MSI testing in patients with mCRC [22]. The Brazilian Gastrointestinal Tumors Group recommends having the same molecular profile in the metastatic setting [23]. In Chile, Mexico, Peru and Uruguay, local guidelines created by government entities or expert consensus follow international recommendations [22, 24–26]. *BRAF* molecular testing is generally required once patients are in the metastatic setting.

Access to tools for BRAF molecular testing is limited across LA. Real-time polymerase chain reaction (RT-PCR) and next-generation sequencing (NGS) are available, but only via clinical trials because of their high cost (Table 1). Despite the higher accuracy of RT-PCR, some local laboratories across LA still apply Sanger Sequencing, although the accuracy may be suboptimal. Pharmaceutical companies sponsor RAS and BRAF testing for anti-EGFR therapeutic management across LA to support patient access to precision medicine diagnostics.

### Value of early BRAF-mutation status in mCRC in therapeutic decision-making and understanding its importance

Early *BRAF* status detection is critically important from a prognostic, predictive and therapeutic standpoint. Before starting first-line therapy in mCRC patients, international guidelines recommend *BRAF*, *RAS* and MSI testing.

*BRAF*-mutated tumors, particularly those harboring a V600E mutation, are associated with significantly poorer OS than *BRAF*-V600 wild-type tumors (median 10.4 versus 34.7 months), and a higher rate of peritoneal and distant lymph node metastasis [27]. Other randomised clinical trials also reported the negative impact of *BRAF* mutations [7, 28, 29]. Another advantage of identifying the *BRAF* status early is connected to MSI. If a *BRAF* mutation is found in dMMR/MSI tumors, Lynch syndrome can mostly be ruled out. Thus, determining *BRAF status* is of diagnostic and therapeutic relevance and helps differentiate somatic (sporadic) from germline (hereditary) dMMR [30–32].

RAF protein dimerization and sensitivity to BRAF and MEK inhibitors support the proposed molecular classification for *BRAF* mutations; Class I (including V600E) mutations have BRAF activity as monomers. Class II mutations are active only as dimers. Both are RAS-independent in activating the MAPK pathway. Class III *BRAF* mutations are not intrinsically active, requiring coexisting *RAS* mutations to activate the MAPK system. *RAS* (*KRAS* and *NRAS*) mutations in the RAS-RAF-MAPK and PI3K-AKT-mTOR signaling pathways are recognised as anti-EGFR resistant biomarkers [27]. The non-V600E-*BRAF* mutations represent less than 5% of *BRAF* mutations. They confer a similar prognosis as *RAS/BRAF* wild-type tumors. Some reports suggest that non-*BRAF*-*V600E*-mutated tumors might benefit from anti-EGFR therapy.

According to the European Society for Medical Oncology (ESMO) guidelines [20], *BRAF-V600E* characterization is vital for prognosis and treatment. First-line treatment includes chemotherapy with the classic scheme of FOLFOX or FOLFIRI plus a biologic, preferably anti-angiogenic therapy with bevacizumab. Different meta-analyses evaluating the predictive role of *BRAF* mutations show the same results in resistance to EGFR inhibitors [33].

In this panel’s experience, *BRAF-V600E* testing in mCRC combined with full RAS testing is needed to provide prognostic and predictive information. Sanger sequencing, RT-PCR and NGS are available across LA, but access depends on patient insurance and reimbursement. Therefore, programs sponsored by the pharmaceutical industry often provide testing to patients who are candidates for anti-EGFR therapies. LB is available in some countries; however, not all countries have granted market approval. *BRAF-V600E* testing is rarely performed in countries that do not have access to targeted therapies. This may be because it could be unethical to test for a condition when therapy is not accessible. However, this action may preclude patients from seeking care abroad or enrolling in clinical trials.

### MSI: predictive marker of chemotherapy effectiveness in BRAF-V600E mCRC

While BRAF-V600E mutations are consistently associated with poor prognosis in mCRC, the impact of MSI-H status is more nuanced. It can vary depending on the stage of the disease and available treatment options. The combination of both factors creates a complex clinical scenario that requires careful consideration in treatment planning and prognostication [24–26].

MSI-H status in early-stage CRC (stages II and III) is generally considered a favorable prognostic factor [34]. The dMMR/MSI-H CRCs account for 15%–20% of stage II and III CRCs, representing only approximately 4% of mCRC cases; a lower frequency indicates the weakened capacity for dMMR CRCs to develop metastases [35]. When *BRAF-V600E* mutations and MSI-H status occur together, which happens in approximately 30% of *BRAF-V600E*-mutated mCRC tumors, it is generally associated with poor OS in stage III CRC [36]. However, MSI-H status in mCRC is associated with improved response to immune checkpoint blockade, potentially improving outcomes for these patients [36]. Pembrolizumab, an ICI, is indicated for treating MSI-H/dMMR mCRC patients [37]. The combination of *BRAF-V600E* mutation and MSI status affects the efficacy of targeted therapies. Encorafenib (BRAF inhibitor) combined with cetuximab (anti-EGFR antibody) is indicated for previously treated *BRAF-V600E*-mutant mCRC patients [37]. In some cases, patients with both BRAF V600E mutation and MSI-H status may show atypical response patterns to different treatment sequences [38].

**Figure 2.**
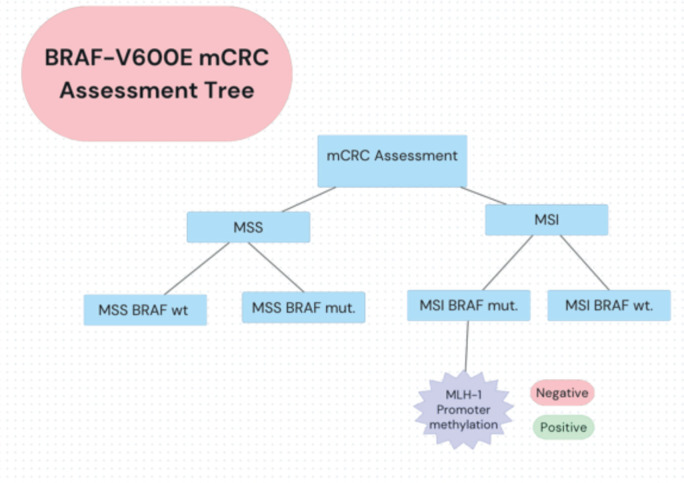
BRAF-V600E mCRC assessment decision tree. Oncologists in LA apply a summarised decision algorithm to detecting MSS/MSI status and mutational status among mCRC patients.

The unique characteristics of BRAF V600E-mutated, MSI-H mCRC have prompted further research. The SEAMARK study evaluates the combination of pembrolizumab with encorafenib and cetuximab versus pembrolizumab alone in first-line treatment for BRAF V600E-mutant and MSI-H/dMMR mCRC [37, 39, 40]. Novel approaches include triple combinations of BRAF inhibitors, anti-EGFR antibodies and immune checkpoint inhibitors specifically for the MSI-H population [28, 38]. An assessment tree for diagnosing BRAF-V600 mCRC is depicted in Figure 2.

The results of these diagnostic tests are essential to guiding the therapeutic management of mCRC. Regarding evidence on the impact of immunotherapy in patients with MSI/dMMR and *BRAF-V600E* mutated tumors, some patients with these traits were included in the pembrolizumab and nivolumab plus or minus ipilimumab trials. In CheckMate 142, patients with histologically confirmed mCRC and MSI-H/dMMR were treated in one of three cohorts: one for first-line patients receiving nivolumab plus ipilimumab, and the other two for patients with at least two prior lines of treatment (2L), one in monotherapy with nivolumab and the other nivolumab plus ipilimumab.

The ORR in all three cohorts was encouraging, with no significant differences from those observed in the wild-type *BRAF* subgroup: 82% versus 62% in the first-line cohorts, 42% versus 45% in the nivolumab-alone 2L cohort and 70% versus 61% in the nivolumab-ipilimumab 2L group. Consistent with these data, there were no differences in the primary endpoint of PFS between the *BRAF* wild-type (HR 0.50) and *BRAF-V600E* (HR 0.48) groups in the Keynote 177 trial, with 77 patients carrying the *BRAF-V600E* mutation [47].

## Therapeutic decision-making for the management of BRAF-V600E mCRC in LA

Clinical trial results and regulatory approvals impact recommended treatment decisions for managing *BRAF-V600E* mCRC in LA. In 2015, the phase 3 TRIBE study evaluated the chemotherapy intensification with FOLFOXIRI plus bevacizumab compared with FOLFIRI plus bevacizumab as a first-line treatment for mCRC, and 7% (28/391) of *BRAF*-mutated patients were identified. Results of this *BRAF*-mutated subgroup indicated a better median OS for the FOLFOXIRI plus bevacizumab treatment, 19 versus 10.7 months, HR 0.54 as well as a median PFS of 7.5 versus 5.5 months, and an ORR of 56 versus 42 [48].

Since those results were published, the triplet chemotherapy regimen plus bevacizumab became the standard first-line choice for this *BRAF*-mutated subgroup of patients. However, the TRIBE2 study compared upfront FOLFOXIRI plus bevacizumab with FOLFOX plus bevacizumab followed by FOLFIRI plus bevacizumab after disease progression did not reproduce these findings, and they reported no increased benefit from the intensified approach [48]. This might be explained by the different comparator group that was an oxaliplatin-based doublet instead of an irinotecan-based doublet as in TRIBE or by *BRAF*-mutated clinical heterogeneity.

A meta-analysis of five studies also showed no increased benefit from the intensified approach of FOLFOXIRI plus bevacizumab in *BRAF*-mutated patients. Thus, using FOLFOX plus bevacizumab can be an upfront option, leaving the triplet combination for patients needing a response, including those with the potential to convert to resectability and those with good performance status scores [48].

There are controversies concerning the use of anti-EGFR agents in *BRAF*-mutated patients. One meta-analysis of nine phase-3 trials and one phase-2, with 463 colorectal *BRAF*-mutated patients in first- and second-line treatments, showed no benefit of the anti-EGFR drug, either for median PFS (HR 0.88, *p* = 0.33) or median OS (HR 0.91, *p* 0.63), and then recommended an anti-VEGF based treatment for this group [33]. However, another meta-analysis of seven randomised trials found inadequate evidence that *BRAF*-mutated patients benefit differently from anti-EGFR medicines than *BRAF* wild-type individuals [49]. In addition, an individual patient data meta-analysis of the randomised trials from the ARCAD database found no evidence of additional efficacy of the anti-EGFRs in these patients [50].

The BEACON trial randomised 665 *BRAF-V600E* mCRC patients who had progressed after one or two previous regimens of encorafenib (a BRAF inhibitor), binimetinib (a MEK-inhibitor) and cetuximab (an anti-EGFR), known as the triplet arm; encorafenib; cetuximab, known as the doublet arm; or cetuximab and irinotecan-based chemotherapy, known as the control arm. Regarding the primary endpoint median OS, the findings showed 9 versus 5.4 months, favoring the triplet over the control arm; HR 0.52, *p* < 0.001 and 8.4 versus 5.4 months, favoring the doublet over the control arm; HR 0.60, *p* <0.001; ORRs were 26%, (triplet) 20%, (doublet) and 2% (control) [51]. Thus, targeted therapy in the second line can lead to survival benefits and response rate improvements.

Other options for previously treated patients came from smaller studies, including the SWOG 1406, which started with a phase-1b study combining irinotecan, cetuximab and vemurafenib [52]. Vemurafenib (a BRAF inhibitor) and the combination had a 35% ORR and 7.7 months of median PFS that led to the phase-2 study of irinotecan and cetuximab with or without vemurafenib [39]. The study randomised 106 patients and reported a median PFS benefit of 4.2 versus 2.0 months, HR of 0.50 and a *p* < 0.001, with an ORR of 17% versus 4%, favoring the vemurafenib arm. The crossover rate was high as 42%, and there was no significant median OS difference.

Another preliminary trial evaluated the combination of panitumumab (an anti-EGFR monoclonal antibody) and dabrafenib (a BRAF inhibitor), the combination plus trametinib (a MEK-inhibitor), and panitumumab and trametinib in 142 patients split into three arms. They found an ORR of 10%, 21% and 0%; a median PFS of 3.5, 4.2 and 2.6 months; and a median OS of 13.2, 9.1 and 8.2 months, respectively, but with poor tolerability related to skin toxicity in the triplet arm [53].

After the BEACON trial, the ANCHOR trial tested the same regimen in the first line. ANCHOR is a single-arm phase-2 trial evaluating encorafenib, binimetinib and cetuximab in *BRAF*-V600E-mutant mCRC first-line treatment. It showed a 50% ORR in the first 41 patients [54] and maintained a 47.8% ORR after including 95 new patients, with a median PFS of 5.8 months and a median OS of 17.2 months [55].

New chemotherapy-free, targeted treatments exist for patients with *BRAF*-mutated mCRC. Based on BEACON trial results, the chemotherapy-free therapy of combining encorafenib (a BRAF inhibitor) and cetuximab (an anti-EGFR antibody) was approved by the European Union (EU) in June 2020. This combination is approved for treating adult patients with BRAF-V600E mCRC who have previously received systemic therapy [40].

The ongoing phase 3 BREAKWATER trial has three arms (encorafenib, binimetinib and chemotherapy with mFOLFOX6 or the same target drugs plus chemotherapy with FOLFIRI). Preliminary results show an ORR of 68.4%, with 25.9% of participants experiencing treatment-related serious adverse events (AEs) in the mFOLFOX6 arm versus an ORR of 66.7% and 13.3% in the FOLFIRI arm [40, 56].

Currently, the recommended treatments include a cytotoxic chemotherapy combination (doublet or triplet) plus bevacizumab in the first line and a combination of a BRAF inhibitor (preferably encorafenib) with an anti-EGFR (cetuximab) with or without a MEK-inhibitor (binimetinib) in the second-line, or, if a BRAF inhibitor is unavailable, another doublet plus an anti-VEGF drug. The exception is the concomitant MSI-H status patients, who seem to benefit from ICIs as the first option [57].

In LA, socioeconomic diversity and drug availability are additional barriers to treatment. The Central America and Caribbean consensus on the management of mCRC recommended a first-line doublet (FOLFOX or FOLFIRI) or triplet (FOLFOXIRI) with an anti-VEGF antibody for patients with *BRAF*-mutated non-resectable cancer and for whom debulking surgery can be objective [58].

In the 2022 guidelines, the Brazilian Society of Clinical Oncology (SBOC) stated the preferred options for first-line treatment in the *BRAF-V600E*-mutated mCRC included doublet (mFOLFOX6 or FOLFIRI) or for young patients with aggressive disease and no comorbidities, triplet (FOLFOXIRI) plus or minus bevacizumab. The SBOC recommends including a BRAF inhibitor (encorafenib plus cetuximab plus or not binimetinib or the combination of vemurafenib plus irinotecan plus cetuximab) in second-line treatment [59].

The combination’s safety profile is similar to that of the individual medications. For anti-VEGF, the main AEs are related to thrombosis, bleeding or hypertension. The most common AEs for the chemotherapy combination are nausea, diarrhea, peripheral neuropathy (due to oxaliplatin), leukopenia, thrombocytopenia and anemia.

The BEACON trial reported that the most common AEs grade 3 or higher in the triplet arm were gastrointestinal-related ones, including diarrhea (10%), nausea (5%), vomiting (4%), abdominal pain (6%) and skin-related events (acneiform dermatitis, 2%). All grade headache (19%), musculoskeletal pain (12%), arthralgia (19%) and myalgia (13%) occurred more frequently in the doublet-therapy group (BRAF- and EGFR-inhibitors). This counterintuitive disparity in toxicity may be explained by MEK inhibition’s capacity to attenuate BRAF inhibition’s harmful effects. In this trial, the rates of AEs were similar in the triplet and the doublet arm but higher in the control arm [40].

The ANCHOR trial showed similar AEs as the BEACON trial, besides an acute kidney injury rate of grade 3 or more (5.3%). The SWOG 1406 trial showed higher grade 3 and 4 AEs in the vemurafenib arm, especially neutropenia (30 versus 7%), anemia (13 versus 0%) and nausea (19 versus 2%), along with more diarrhea and fatigue. Side effects are specific to each chemotherapy agent or a class impact for targeted treatments. Still, they are manageable, as reported in most trials [54].

### Early prediction of BRAF-mutational status for informing stronger treatment decisions

The treatment of *BRAF-V600E* mCRC has improved over the last decade because of the parallel evolution of preclinical and clinical knowledge. Cancer immune therapy with ICIs has also shown improved results in MSI tumors. However, the true challenge in chemotherapeutics is represented by patients with *BRAF-V600E* dMMR tumors. Current clinical studies combining targeted medicines and checkpoint inhibitors are expected to broaden the therapy range for this subtype of CRC under the precision oncology paradigm. Overall, the recent approval of the combination of cetuximab and encorafenib represents a step forward for treating *BRAF-V600E* mCRC. Due to the disease’s aggressiveness, only 50% of patients reach second-line therapy. Because of this prognostic impact, it is crucial to consider early enrollment in ongoing clinical trials of all patients with *BRAF-V600E* mCRC.

### BRAF-mutated mCRC and international standards of care

Local guideline availability for testing and treating *BRAF*-mutated tumors is limited in many regions, including LA. As a result, physicians often use the international standards of care for *BRAF*-mutated mCRC. While most countries in LA have access to some chemotherapy options for mCRC, access to novel therapeutics beyond bevacizumab is more limited. LA treatment options typically involve doublet or triplet chemotherapy regimens combined with bevacizumab and are not consistently available in all countries. The recent approval of encorafenib by the USFDA and the European Medicines Agency in April 2020 has not yet resulted in widespread access to this drug for non-clinical trial patients in LA. While encorafenib is approved in Argentina, Brazil and Chile, it is not widely available. In addition to the lack of guidelines for testing and treatment, the availability of CRC treatment is drastically limited in most LA countries, except for Brazil. Physicians often use international guidelines like those from the National Comprehensive Cancer Network (NCCN), Clinical Practice Guidelines in Oncology, the American Society of Clinical Oncology (ASCO) and the ESMO.

## Discussion

This panel recognises the prodigious challenge of accessing evidence-based prevention, diagnosis and treatment strategies for mCRC in LA. Molecular profiling and novel predictive biomarkers add complexity. Equanimous access to molecular diagnostics and optimum medical and surgical techniques must be promoted and ensured [60]. The region’s lack of access to effective screening programs and evidence-based surgical and clinical treatments is omnipresent. This context should be faced and prioritised as a *sine qua non* condition for improving patient care and reducing CRC mortality in LA over the next few decades (Table 2).

The *BRAF*-mutated advanced CRC is a rapidly evolving field in modern oncology. The NCCN [61], ASCO [62] and ESMO [20] have specific recommendations for *BRAF*-mutated CRC patients in their advanced CRC Guidelines.

To improve outcomes and prognosis for *BRAF*-mutated mCRC patients in LA, the panel proposes:

1. Local guidelines to manage mCRC patients are necessary to contextualise cancer care to specific LA conditions. This panel favors the creation of local cancer care guidelines and for these guidelines to be adopted within LA national and local oncology societies to help clinical oncologists provide the best care to each patient V-A.
2. Multidisciplinary boards to deliver better individual decisions and care to patients with mCRC. The panel recommends that each mCRC patient care plan be discussed within multidisciplinary boards of clinical and surgical specialists in gastrointestinal cancer V-A.
3. Tumor tissue genotyping for all patients with mCRC. This can include *BRAF* mutations assayed individually or as part of a multiple genes panel. (I-A) If no tissue is available, an LB test could be used (II-B). Several validated platforms (PCR, NGS and immunohistochemistry) can perform the test. The test can be done using the primary tumor and/or the metastasis. Since there is no predictive role of *BRAF* testing results in localised CRC, the test should be routinely performed on relapsed/unresectable or stage IV disease I-A.
4. First-line treatment for *BRAF*-mutated pMMR tumors should include a combination of irinotecan or oxaliplatin-based fluoropyrimidine doublets or triplets (in patients with suitable functional and performance status) with bevacizumab [63] (II-B). Patients with dMMR tumors should receive pembrolizumab as a first-line treatment [47] II-A.
5. After disease progression, patients should receive second-line treatment of a BRAF inhibitor plus EGFR inhibition (encorafenib + cetuximab) (I–A) [40]. Triplet regimens with the addition of a MEK-inhibitor to BRAF- and EGFR-inhibitors are under investigation and should not be routinely recommended. Patients with *BRAF*-mutated tumors should not receive anti-EGFR monoclonal antibodies unless given with BRAF inhibition. Other than V600E-*BRAF* mutations, the best treatment is yet to be defined [64, 65] II-B.
6. Citorreductive surgeries or metastasis resections should be performed according to the multidisciplinary board’s judgments, and mutation identification should not cause delaying curative procedures from oligometastatic *BRAF-V600E* tumors V-A.

## Conclusion

In conclusion, treating patients with BRAF*-V600E* mCRC remains a challenge throughout LA, with opportunities for improvement in accessing diagnosis and treatment, and participating in clinical trials. While most countries in LA have access to some chemotherapy options for mCRC, access to novel therapeutics beyond bevacizumab is limited. *BRAF-V600E* mCRC treatment has been steadily improving because of advances in preclinical and clinical research and the emergence of cancer immune therapies with ICIs that have shown promising results in MSI tumors. However, challenges to accessing these new therapies across the region remain. Besides standard therapies for *BRAF*-*V600E* mCRC, which are limited, new chemotherapy-free targeted options have emerged in adult patients with *BRAF-V600E* mCRC who have received prior systemic treatment. However, due to delays in regulatory approvals in some LA countries, encorafenib is not yet widely available.

## Conflicts of interest

The authors declare no conflicts of interest.

## Author contributions

AKC, CB, MACG, JMOC, JCSPV, GM: Validation, formal analysis, writing – original draft.

AMJ: Visualization, project administration, writing – review and editing. MRR: Conceptualization, methodology, writing – review and editing.

GS: Resources, supervision, visualization, writing – review and editing.

## Disclosure of results at a meeting

The results of this paper were presented as a poster at the 25th World Congress on Gastrointestinal Cancer on 28 June 2023.

## Figures and Tables

**Table 1.** *BRAF-V600E* for mCRC testing in Argentina, Brazil, Colombia and Mexico.

	Sanger sequencing	Pyrosequencing	NGS	LB
Market approval	Not required	Not required	EU: No; USA: Partly	Yes
Indication	Multiple	Multiple	Multiple	Multiple
Lab detection time	2–3 days	2 days	2–4 days	1 day
Test availability by country				
Argentina	x	x	x	
Brazil	x	x	x	x
Colombia	x	x	x	
Mexico	x	x		

Legend: NGS: Next generation sequencing, EU: European Union, USA: United States of America, x: Available

**Table 2.** Panel recommendations summary.

Panel statement	Evidence level-recommendation strength
**1. Local guidelines to manage advanced mCRC patients should be created.**	V-A
**2. Multidisciplinary Boards should be created to discuss and manage patient care plans.**	V-A
**3. All patients with mCRC should have tumor tissue genotyped for *BRAF* mutations individually or as part of a multiple genes panel.**	I-A
**4. The test should be performed in the primary tumor and/ or in the metastasis and routinely performed in relapsed/ unresectable or metastatic disease (stage IV)**	I-A
**5.** Liquid biopsies **are acceptable in the absence of tissue availability to use for *BRAF* testing.**	II-B
**6. First-line systemic treatment to *BRAF-V600E* mutated pMMR tumors should include oxaliplatin/ irinotecan – fluoropyrimidine as doublets or triplets plus bevacizumab.**	II-B
**7. Patients with dMMR BRAF-mutated tumors should receive pembrolizumab as a first-line systemic treatment.**	II-A
**8. Second-line systemic treatment should include BRAF inhibitors + Anti-EGFR combinations**	II-A
**9. Non-*V600E-BRAF* mutations can have different clinical characteristics and/or prognoses; the appropriate treatment is yet to be defined.**	II-B
**10. Curative or citorreductive surgical procedures should be considered in the multidisciplinary board’s decisions. *BRAF*-mutational status should prevent interventions in patients with curative potential.**	V-A

Legend: **Level of evidence**

I At least one large, well-conducted randomized control trial or meta-analyses of such trials

II Randomized control trials with suspicion of bias or meta-analyses of such trials

III Prospective cohort studies

IV Retrospective cohort studies case-control studies

V Studies without a control group, case reports, and experts’ opinions

**Strength of recommendation**

A Strongly recommended

B Generally recommended

C Optional

D Generally, not recommended

E Never recommended
